# A new-generation of *Bacillus subtilis* cell factory for further elevated *scyllo*-inositol production

**DOI:** 10.1186/s12934-017-0682-0

**Published:** 2017-04-21

**Authors:** Kosei Tanaka, Ayane Natsume, Shu Ishikawa, Shinji Takenaka, Ken-ichi Yoshida

**Affiliations:** 1 Organization of Advanced Science and Technology, Kobe University, Kobe, Japan; 2 Graduate School of Agricultural Science, Department of Agrobioscience, Kobe University, Kobe, Japan; 3 Graduate School of Science, Technology and Innovation, Department of Science, Technology and Innovation, Kobe University, 1-1 Rokkodai-cho, Nada-ku, Kobe, Hyogo 657-8501 Japan

**Keywords:** *Bacillus subtilis*, *scyllo*-inositol, *myo*-inositol, Bioconversion, Alzheimer’s disease

## Abstract

**Background:**

A stereoisomer of inositol, *scyllo*-inositol (SI), has been regarded as a promising therapeutic agent for Alzheimer’s disease. However, this compound is relatively rare, whereas another stereoisomer of inositol, *myo*-inositol (MI) is abundant in nature. *Bacillus subtilis* 168 has the ability to metabolize inositol stereoisomers, including MI and SI. Previously, we reported a *B. subtilis* cell factory with modified inositol metabolism that converts MI into SI in the culture medium. The strain was constructed by deleting all genes related to inositol metabolism and overexpressing key enzymes, IolG and IolW. By using this strain, 10 g/l of MI initially included in the medium was completely converted into SI within 48 h of cultivation in a rich medium containing 2% (w/v) Bacto soytone.

**Results:**

When the initial concentration of MI was increased to 50 g/l, conversion was limited to 15.1 g/l of SI. Therefore, overexpression systems of IolT and PntAB, the main transporter of MI in *B. subtilis* and the membrane-integral nicotinamide nucleotide transhydrogenase in *Escherichia coli* respectively, were additionally introduced into the *B. subtilis* cell factory, but the conversion efficiency hardly improved. We systematically determined the amount of Bacto soytone necessary for ultimate conversion, which was 4% (w/v). As a result, the conversion of SI reached to 27.6 g/l within 48 h of cultivation.

**Conclusions:**

The *B. subtilis* cell factory was improved to yield a SI production rate of 27.6 g/l/48 h by simultaneous overexpression of IolT and PntAB, and by addition of 4% (w/v) Bacto soytone in the conversion medium. The concentration of SI was increased even in the stationary phase perhaps due to nutrients in the Bacto soytone that contribute to the conversion process. Thus, MI conversion to SI may be further optimized via identification and control of these unknown nutrients.

## Background

There are nine possible stereoisomers of inositol (1,2,3,4,5,6-cyclohexanehexol). One of the stereoisomers, *myo*-inositol (MI), is the most abundant in nature, while others are relatively rare [1]. It has been reported that some of the inositol stereoisomers have specific health-promoting activities. For example, d-*chiro*-inositol (DCI) is a promising investigational drug candidate for the treatment of type 2 diabetes; interestingly, administration of DCI to diabetic rats accelerated glucose disposal and sensitized insulin action [2]. In addition, both DCI and MI are effective in ameliorating metabolic aspects of polycystic ovary syndrome [3–7]. Another stereoisomer, *scyllo*-inositol (SI), has been regarded as a promising therapeutic agent for Alzheimer’s disease as demonstrated via oral administration of SI to a mouse model of Alzheimer’s disease, which inhibited amyloid β protein (Aβ) aggregation, attenuated Aβ-induced impairments of spatial memory, reduced cerebral Aβ pathology, and decreased the rate of mortality [8]. Therefore, SI has received a fast-track designation from the US Food and Drug Administration for treatment of mild to moderate Alzheimer’s disease and is intended to progress into phase III development [9].

**Fig. 1:**
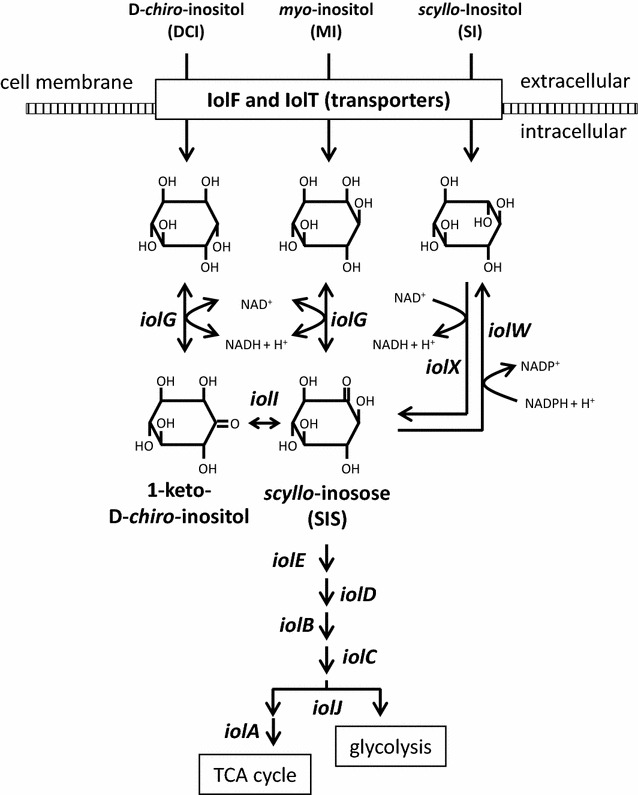
Inositol metabolism in *Bacillus subtilis*

*Bacillus subtilis* is able to grow in a minimal medium containing DCI, MI, and SI as its sole carbon source, respectively (Fig. 1), and the gene set necessary for their utilization has been characterized [10]. In the first step, DCI is transported into the cells mainly by IolF and secondarily by IolT transporters, whereas MI and SI are transported mainly by IolT and inefficiently by IolF [10, 11]. These inositol stereoisomers are metabolized continuously by a number of enzymes encoded by the *iolABCDEFGHIJ* operon [12]. Transcription of the operon is suppressed by the IolR transcriptional repressor, whose gene is located immediately upstream of the operon with divergent orientation, and induced by addition of the inositol stereoisomers into medium [13, 14]. Once DCI and MI are transported into the cells, the inositol stereoisomers are converted to 1-keto-d-*chiro*-inositol and *scyllo*-inosose (SIS), respectively, by the IolG enzyme with NAD^+^ reduction. The former is isomerized to the latter by IolI (Fig. 1). SIS is metabolized sequentially by IolE, IolD, IolB, IolC, IolJ, and IolA, resulting in intermediates that enter glycolysis and the TCA cycle as dihydroxyacetone phosphate and acetyl-CoA, respectively. While SI is converted into SIS by two additional inositol dehydrogenases, IolX and IolW, with NAD^+^ and NADP^+^ reduction, respectively [14]. In vivo and in vitro analyses have revealed that IolX plays the major physiological role in SI catabolism, whereas IolW efficiently reduces SIS into SI via oxidation of NADPH. SIS is degraded further via the metabolic pathway described above.

**Fig. 2:**
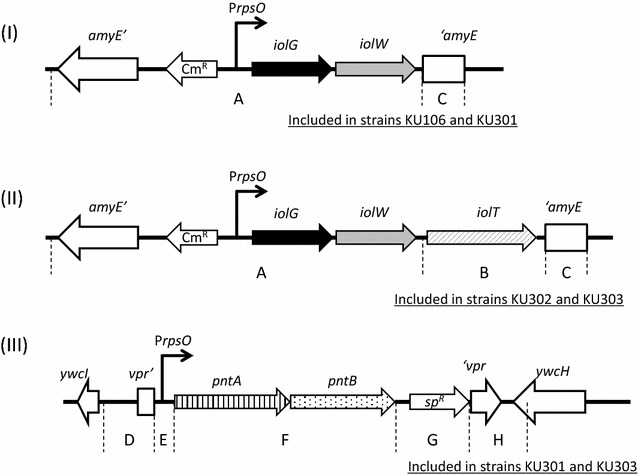
Organization of simultaneous overexpression of IolG-IolW (**I**), IolG-IolW- IolT (**II**), and PntA-PntB (**III**). Overexpression of IolG and IolW was constructed in a previous study [15] (**I**). For simultaneous overexpression of IolG, IolW, and IolT, PCR fragments covering regions A + B + C were ligated by recombinant PCR and integrated into the *amyE* locus by a double crossover event (**II**). For overexpression of PntA and PntB, PCR fragments covering regions D + E + F + G + H were ligated and integrated into the *vpr* locus by a double crossover event (**III**)

In a previous study, we reported two generations of cell factories to produce SI from MI using genetically modified *B. subtilis* cells [10, 15]. Strain TM039, a prototype of a cell factory, was constructed by deletion of the three genes *iolR, iolX*, and *iolI* and by introduction of a missense mutation *iolE41.* Those modifications were designed to enable constitutive expression of the *iolABCDEFGHIJ* operon, including *iolG*, and to disable dehydrogenation of SI as well as isomerization and dehydration of SIS. Conversion of SI in the strain achieved nearly half of the initial MI amount (10 g/l) after 72 h cultivation, but the other half of MI was consumed [10]. Not only to solve this problem but also to increase the conversion efficiency, strain KU106, a *B. subtilis* second-generation cell factory that contains deletions of *iolR, iolX*, and *iolABCDEFHIJ* and simultaneous overexpression of *iolG* and *iolW,* was constructed (Fig. 2(I)) [15]. Finally, 10 g/l of MI initially contained in the medium was completely converted to SI within 48 h of cultivation. In this study, we noticed that the conversion efficiency was still limited when the initial concentration of MI was increased up to 50 g/l. To overcome this limitation, we constructed a third, new-generation cell factory in which the uptake system of MI and regeneration system of NADPH were enhanced. Furthermore, amount of Bacto soytone in the conversion medium was increased from 2 to 4% (w/v) because it has been reported that sufficient Bacto soytone is necessary for ultimate conversion [15].

## Results

### Bioconversion efficiency of strain KU106

**Fig. 3:**
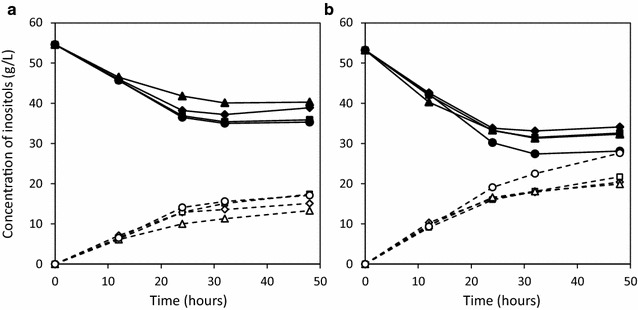
Time course of SI production and MI consumption. Bioconversion was performed by strains KU106 (P*rpsO*-*iolG-iolW*; *diamonds*), KU301 (P*rpsO*-*iolG-iolW,* P*rpsO*-*pntA-pntB*; *squares*), KU302 (P*rpsO*-*iolG*-*iolW*-*iolT*; *triangles*), and KU303 (P*rpsO*-*iolG-iolW-iolT,* P*rpsO*-*pntA-pntB*; *circles*) in the conversion medium containing 2% (w/v) (**a**) and 4% (w/v) (**b**) of Bacto soytone, respectively. Concentration of MI (*continuous lines*) and SI (*dotted lines*) in the culture medium is shown. The experiments were repeated independently at least three times with similar results, and the representative data sets are shown

Strain KU106 (Δ*iolABCDEF* Δ*iolHIJ* Δ*iolX* Δ*iolR, amyE::*P*rpsO*-*iolG*-*iolW* (*cat*)) was able to convert all of MI into SI in the conversion medium containing 2% (w/v) Bacto soytone within 48 h of cultivation when 10 g/l of MI was added to the medium [15]. For further conversion, concentration of MI in the medium was increased to 50 g/l, and the conversion was performed using strain KU106. The growth were almost similar between the two conditions (data not shown), because this strain is unable to metabolize MI as mentioned above. Even the amount of MI was increased, the resulting amount of SI was no more than 15.1 g/l in the medium containing 2% (w/v) Bacto soytone (Fig. 3a). To identify the rate-limiting factor for the conversion, we attempted further genetic modification. In strain KU106, two-step reactions, MI to SIS catalyzed by IolG with NAD^+^ reduction and SIS to SI by IolW with NADPH oxidization (Fig. 1), have already been enhanced by simultaneous overexpression of IolG and IolW, but regeneration of the cofactors has not been enhanced yet. NAD^+^ necessary for the first reaction is supposed to be easily regenerated by respiration because *B. subtilis* is a typical aerobic bacterium, but the homologue of the transhydrogenase gene for NADPH production does not exist in this bacterium. Therefore, we considered that the lack of efficient regeneration system of NADPH in *B. subtilis* as one of the reasons for the limited conversion. In addition, we expected that uptake of MI could also be a rate-limiting factor for the conversion.

### Overexpression of transhydrogenase

In *Escherichia coli,* NADPH is regenerated not only by metabolic reactions such as the TCA cycle and pentose phosphate pathway but also by membrane-integral nicotinamide nucleotide transhydrogenase, which drives the reduction of NADP^+^ via the oxidation of NADH [16, 17]. In addition, it has been previously demonstrated that amplification of the transhydrogenase systems has been employed for NADPH-dependent bioprocesses in *E. coli* with success [17]. On the other hand, and as mentioned above, *B. subtilis* does not possess the homologue of transhydrogenase gene, though the enzymatic activity was detected in crude cell extracts of exponentially grown *B. subtilis* [18, 19]. To supply enough NADPH for the conversion, we attempted overexpression of *E. coli* transhydrogenase in the strain. There are two types of transhydrogenase in *E. coli* including membrane-integral nicotinamide nucleotide transhydrogenase (PntAB) and soluble transhydrogenase (UdhA) [17]. The former catalyzes NADH and NADP^+^ to NAD^+^ and NADPH (NADPH production), whereas the latter catalyzes NAD^+^ and NADPH to NADH and NADP^+^ (NADH production). For the enhancement of NADPH production, PntAB was chosen and overexpressed (Figs. 2(III), 3a, KU301). As a result, conversion efficiency in strain KU301 was slightly increased (17.1 g/l SI) compared to that in strain KU106 (15.1 g/l SI) (Refer to Fig. 3a). This result indicated that deficiency of NADPH was one of the rate-limiting factors for the conversion and other rate-limiting factors still existed. As uptake system of MI was thought to be one of them, we attempted overexpression of MI transporter.

### Overexpression of the MI transporter

IolF and IolT have been reported as minor and major transporters of MI, respectively, in *B. subtilis* [11]. As IolF alone is unable to support the growth in minimal medium containing MI as the sole carbon source, IolT was chosen as the candidate to enhance the MI uptake system. The overexpression system of IolT was constructed by introducing *iolT* gene downstream of the *iolG*-*iolW* overexpression system (Fig. 2(II)), and then the conversion was performed. However, the conversion efficiency was not changed in this strain (Fig. 3a, strain KU302), indicating that ability to uptake MI was not the bottleneck for the conversion under this condition. As one possibility, MI was not efficiently converted after transport into the cells. Actually, enough NADPH was necessary for the efficient conversion in strain KU106 as shown above. To confirm the possibility, PntAB overexpression was introduced into the strain (Fig. 3a, strain KU303), and the conversion was performed. Even when both overexpression systems were combined, the conversion efficiency in the strain was equivalent to that in strain KU301. These results indicated that at least enhancement of NADPH production was effective for the conversion, but MI transporter was not under this condition. For further conversion, we next investigated culture condition.

### Culture conditions enabling more efficient conversion

**Fig. 4:**
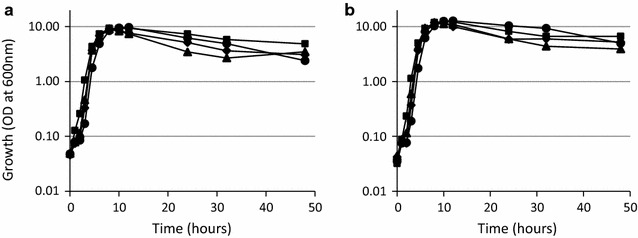
Cell growth in the conversion medium. Cell growth of the strains KU106 (P*rpsO*-*iolG*-*iolW*; *diamonds*), KU301 (P*rpsO*-*iolG*-*iolW,* P*rpsO*-*pntA*-*pntB*; *squares*), KU302 (P*rpsO*-*iolG*-*iolW*-*iolT*; *triangles*), and KU303 (P*rpsO*-*iolG*-*iolW*-*iolT,* P*rpsO*-*pntA*-*pntB*; *circles*) in the conversion medium containing 2% (w/v) (**a**) and 4% (w/v) (**b**) of Bacto soytone is shown. The experiments were repeated independently at least three times with similar results, and the representative data sets are shown

As reported in a previous study, when 2% (w/v) Bacto soytone contained in the conversion medium was reduced, SI production was impaired significantly in strain KU106, although no severe effect on cell growth was observed [15]. Therefore, we tested the conversion in the medium containing a higher concentration of Bacto soytone, such as 4% (w/v), using the strains mentioned above. As expected, production of SI was increased to 20.4, 21.7, 19.9, and 27.6 g/l in strains KU106, KU301, KU302, and KU303, respectively (Fig. 3b), however the growth of the strains was similar between 2 and 4% (w/v) Bacto soytone medium (Fig. 4). Especially, the conversion in strain KU303 reached almost half of the initial MI (50 g/l) in the conversion medium. In this study, the conversion efficiency from MI to SI was able to be improved from 15.1 to 27.6 g/l/48 h by using strain KU303 and increasing the amount of Bacto soytone in the conversion medium. Interestingly, even concentration of Bacto soytone in the medium was increased to 8% (w/v), the conversion efficiency was not improved compared with that in 4% (w/v) Bacto soytone medium (data not shown) though the final optical density in the former medium was almost twice higher than that in the latter one. These results indicated that nutrients included in Bacto soytone was also important factor for the conversion, and the optimal amount of Bacto soytone was 4% (w/v) in strain KU303.

## Discussion

In this study, it was proven that enhancement of regeneration system of NADPH and MI uptake system, namely overexpression of PntAB and IolT respectively, was effective for the conversion from MI to SI when the strain (KU303) was cultivated in the conversion medium containing 4% (w/v) Bacto soytone, and the conversion efficiency reached to 27.6 g/l/48 h (Fig. 3b). Indeed, effectiveness of overexpression of transhydrogenase for NADPH-dependent bioprocesses has been reported in *E. coli* and *B. subtilis* [17, 20]. However, such enhanced conversion was not significantly observed when there was either overexpression of PntAB or IolT (strains KU301 and KU302, Fig. 3b). This meant that both of NADPH and MI were insufficient for the conversion in strain KU106 under this condition. As it has been reported that NADPH is the driving force of most biosynthetic enzymatic reactions such as biosynthesis of lipids and DNA [16], probably catabolically produced NADPH was consumed preferentially for those reactions in the strain. When NADPH was additionally supplied, the reaction from MI to SI smoothly proceeded, resulting in improvement of the conversion efficiency by enhancement of MI uptake system.

On the other hand, overexpression of IolT (strain KU302 and KU303) did not contribute to the improvement of the conversion even PntAB was overexpressed in the medium containing 2% (w/v) Bacto soytone. This is probably because the redox imbalance was caused by the excess NADPH in this culture condition. For the isobutanol production in *B. subtilis*, *udhA* of *E. coli* encoding transhydrogenase, which catalyze NAD^+^ and NADPH to NADH and NADP^+^, was overexpressed to keep redox balance in the strain which produce excess NADPH, and it resulted in enhancement of isobutanol production [20]. By using less strong promoter for the expression of PntAB in strain KU303, it might be possible to maintain proper redox balance, resulting in improvement of the conversion efficiency in this culture condition.

The difference between two culture conditions suggests that nutrient(s) included in Bacto soytone, such as amino acids, altered the metabolism and consequently changed the compounds necessary for improving the conversion. Indeed, it has been reported that addition of glutamate into synthetic medium containing glucose as a carbon source increased fluxes in the pentose phosphate pathway, which caused overproduction of NADPH, and resulted in effective cellulase production in genome-reduced *B. subtilis* strain MGB874 [21].

Interestingly, maximum conversion efficiency was increased in all four strains when concentration of Bacto soytone was increased to 4% (w/v). Especially, the concentration of SI was vigorously increased in the stationary phase (12–48 h). This may be due to cells being well maintained in the stationary phase by nutrient(s) included in Bacto soytone. However, even concentration of Bacto soytone was increased to 8% (w/v), the conversion efficiency was not improved compared with that in 4% (w/v) Bacto soytone medium in strain KU303 (data not shown). Instead, the final optical density at 600 nm (OD_600_) in 8% (w/v) Bacto soytone medium reached to 25.4, which was almost twice higher than that in 4% (w/v) Bacto soytone medium. Probably, additional nutrient(s) was consumed mostly by the growth.

Future studies are warranted to identify these nutrients and/or metabolic factors and investigate the means to control such nutrients to see if conversion efficiency can be further enhanced. Although uptake systems of inositol stereoisomers such as MI, SI, and DCI in *B. subtilis* have been elucidated [10, 11], the efflux system of SI into the growth medium is still unknown. For further conversion, this unknown system also will be included in the next generation cell factory for SI production.

## Conclusions

We constructed a third-generation of *B. subtilis* cell factory for producing *scyllo*-inositol by introducing an additional overexpression system of *E. coli* transhydrogenase, PntAB, and MI transporter of *B. subtilis*, IolT, into the second-generation cell factory. With this modification, the concentration of SI reached to 27.6 g/l from 50 g/l of MI after 48 h cultivation in the conversion medium containing 4% (w/v) Bacto soytone.

## Methods

### Bacterial strains, culture conditions, and primers

Bacterial strains and oligonucleotide primers used in this study are listed in Tables 1 and 2, respectively. Bacterial strains were maintained in Lysogeny Broth (LB) medium [22]. The antibiotics spectinomycin and chloramphenicol were added to LB medium at final concentrations of 100 and 5 μg/ml, respectively. For the inositol bioconversion, two kinds of medium, which consisted of 0.5% (w/v) Bacto yeast extract (Becton, Dickinson and Co., Sparks, MD, USA), 0.2% (w/v) glucose, 5% (w/v) MI, and 2% (w/v) or 4% Bacto soytone (Becton, Dickinson and Co., Sparks, MD, USA), were used. Strains of *B. subtilis* were inoculated in 50 ml of the bioconversion mediums at an optical density of 0.05 at 600 nm and then incubated at 37 °C with shaking at 180 rpm using a 500 ml flask with baffles.

**Table 1:** Bacterial strains used in this study

Strain	Relevant genotype	Source or reference
168	*trpC2*	Laboratory stock
MYI04	*ΔiolABCDEF ΔiolHIJ ΔiolX ΔiolR*	Laboratory stock
KU106	*amyE::PrpsO-iolG-iolW* (cat) (MYI04 background)	Laboratory stock
KU301	*vpr::PrpsO*-*pntA*-*pntB* (sp) (KU106 background)	This study
KU302	*amyE::PrpsO*-*iolG*-*iolW*-*iolT* (cat) (MYI04 background)	This study
KU303	*vpr::PrpsO*-*pntA*-*pntB* (sp) (KU302 background)	This study

**Table 2:** Oligonucleotide primers used in this study

Name	Sequence (5ʹ → 3ʹ)
a011	CCTTCCAGGGTATGTTTCTC
a014	CGATCAGACCAGTTTTTAATTTGTG
a015	TTAACAAAATTCTCCAGTCTTCACATCG
a125	GATTGCGCGTGCGAAAGAAG
a183	CTTCTTTCGCACGCGCAATCGCCCTGGAATAAGTTTTTGAC
a184	CACAAATTAAAAACTGGTCTGATCGCCAAAAAGAATCCGCAC
a337	GCCCGCAAGAAAAGACGGC
a338	GCTCAGTTAATTCTTTGATGCCATCGATTGGCTTTCTCCCGCTTCC
a339	ATGGCATCAAAGAATTAACTGAGC
a343	CTGTAGACAAATTGTGAAAGG
a344	CTGTTATTGCAATAAAATTAGCC
a345	GGCTAATTTTATTGCAATAACAGCGAAGAAAACAAAAGCTGGCACC
a346	GGCGGGAGAGCAGGGTATTCC
a394	CATCCTGTTTCACCTCCAAATCATATTTAGCCCCAGTTACC
a395	GATTTGGAGGTGAAACAGGATGCGAATTGGCATACCAAGAGAAC
a396	CCTTTCACAATTTGTCTACAGGAGTGACGGCCTCAGCAG

### Mutant construction

Strains MYI04 and KU106 (Fig. 2-(I)) were constructed in the previous study [15]. Strain KU302 (Fig. 2-(II)) overexpressing IolG, IolW, and IolT was constructed as follows. The DNA fragment for region A, containing the C-terminal region of the *amyE* locus; chloramphenicol-resistant cassette; and the *iolG*-*iolW* genes containing the promoter of the *rpsO* gene (P*rpsO*), was amplified by PCR using the primer pairs a011/a125 (Table 2) from chromosomal DNA of strain KU106. Similarly, the DNA fragments for region B containing the *iolT* gene and region C containing the N-terminal region of the *amyE* locus were amplified from chromosomal DNA of strain 168 using the primer pairs a183/a184 and a014/a015 (Table 2), respectively. These three fragments were ligated by recombinant PCR using the outside primers a011 and a015, and then the resulting fragment transformed MYI04 to confer chloramphenicol resistance by a double-crossover event at the *amyE* locus. Strain KU301 and KU303 overexpressing PntA and PntB were constructed as follows (Fig. 2-(III)). Five DNA fragments, the first corresponding to the N-terminal region of the *vpr* locus (Fig. 2-(III); region D), the second for P*rpsO* (region E), the third for *pntA*-*pntB* genes of *E. coli* (region F), the fourth for the spectinomycin resistant cassette of *Staphylococcus aureus* SA2223 (region G), and the fifth for the C-terminal region of the *vpr* locus (region H), were amplified by PCR with the primer pairs of a337/a338, a339/a394, a395/a396, a343/a344, and a345/a346, respectively (Table 2). These five fragments were ligated by recombinant PCR in that order as shown in Fig. 2-(III) using the outside primers a337 and a346. Strains KU106 and KU302 were transformed to be spectinomycin resistant by a double-crossover event at the *vpr* locus to yield KU301 and KU303, respectively.

### Measurement of MI and SI in medium

Bacterial culture was diluted appropriately with pure water when required, and cells were removed from the culture medium by centrifugation. The supernatant was passed through an Amicon Ultra-0.5 ml 3 K Centrifugal Filter (Millipore, Billerica, MA, USA). The eluent was subjected to high-performance liquid chromatography (LaChrom Elite: HITACHI High Technologies, Tokyo, Japan) with refractive index detection using a COSMOSIL Sugar-D column (4.6 × 250 mm) (nacalai tesque, Kyoto, Japan) and maintained at 25 °C with a flow of acetonitrile/water (80/20) at 2 ml/min. The retention time was used to identify the stereoisomers, and refractive index units were used to calculate their concentrations.

## Abbreviations

MI: *myo*-inositol
SI: *scyllo*-inositol
DCI: d-*chiro*-inositol
SIS: *scyllo*-inosose
Aβ: amyloid β protein
